# Prediction of high Ki-67 proliferation index of gastrointestinal stromal tumors based on CT at non-contrast-enhanced and different contrast-enhanced phases

**DOI:** 10.1007/s00330-023-10249-3

**Published:** 2023-09-29

**Authors:** Zhenhui Xie, Shiteng Suo, Wang Zhang, Qingwei Zhang, Yongming Dai, Yang Song, Xiaobo Li, Yan Zhou

**Affiliations:** 1 Department of Radiology, Renji Hospital, School of Medicine, Shanghai Jiao Tong University, Shanghai, China; 2 Division of Gastroenterology and Hepatology, Key Laboratory of Gastroenterology and Hepatology, Ministry of Health, Renji Hospital, School of Medicine, Shanghai Jiao Tong University, Shanghai Institute of Digestive Disease, Shanghai, China; 3 School of Biomedical Engineering, ShanghaiTech University, Shanghai, China; 4 MR Scientific Marketing, Siemens Healthineers Ltd., Shanghai, China

**Keywords:** Radiomics, Tomography, X-ray computed, Gastrointestinal stromal tumors, Ki-67 antigen

## Abstract

**Objectives:**

To evaluate and analyze radiomics models based on non-contrast-enhanced computed tomography (CT) and different phases of contrast-enhanced CT in predicting Ki-67 proliferation index (PI) among patients with pathologically confirmed gastrointestinal stromal tumors (GISTs).

**Methods:**

A total of 383 patients with pathologically proven GIST were divided into a training set (*n* = 218, vendor 1) and 2 validation sets (*n* = 96, vendor 2; *n* = 69, vendors 3–5). Radiomics features extracted from the most recent non-contrast-enhanced and three contrast-enhanced CT scan prior to pathological examination. Random forest models were trained for each phase to predict tumors with high Ki-67 proliferation index (Ki-67>10%) and were evaluated using the area under the receiver operating characteristic curve (AUC) and other metrics on the validation sets.

**Results:**

Out of 107 radiomics features extracted from each phase of CT images, four were selected for analysis. The model trained using the non-contrast-enhanced phase achieved an AUC of 0.792 in the training set and 0.822 and 0.711 in the two validation sets, similar to models trained on different contrast-enhanced phases (*p* > 0.05). Several relevant features, including NGTDM Busyness and tumor size, remained predictive in non-contrast-enhanced and different contrast-enhanced images.

**Conclusion:**

The results of this study indicate that a radiomics model based on non-contrast-enhanced CT matches that of models based on different phases of contrast-enhanced CT in predicting the Ki-67 PI of GIST. GIST may exhibit similar radiological patterns irrespective of the use of contrast agent, and such radiomics features may help quantify these patterns to predict Ki-67 PI of GISTs.

**Clinical relevance statement:**

GIST may exhibit similar radiomics patterns irrespective of contrast agent; thus, radiomics models based on non-contrast-enhanced CT could be an alternative for risk stratification in GIST patients with contraindication to contrast agent.

**Key Points:**

• *Performance of radiomics models in predicting Ki-67 proliferation based on different CT phases is evaluated*.

• *Non-contrast-enhanced CT–based radiomics models performed similarly to contrast-enhanced CT in risk stratification in GIST patients*.

• *NGTDM Busyness remains stable to contrast agents in GISTs in radiomics models*.

**Supplementary Information:**

The online version contains supplementary material available at 10.1007/s00330-023-10249-3.

## Introduction

Gastrointestinal stromal tumors (GISTs) are the most prevalent mesenchymal neoplasms found in the human gastrointestinal tract and arise from interstitial cells of Cajal or their stem cell-like precursors. Distinguishing benign from malignant GISTs has been challenging, even with the aid of morphological studies. Over the years, numerous systems for risk stratification have been established [1, 2]. Previous risk stratification systems primarily relied on pathologic features, although it was difficult to predict the malignancy of tumor based on these features alone [2].

Recently, various gene mutations and immunohistochemical tumor markers have been explored to evaluate the biological behavior of GISTs. Ki-67 is a protein expressed in all phases of cell cycle except the rest phase (G0), and is generally used as a marker for cell proliferation [3]. Ki-67 has been recently used as a prognostic biomarker for a wide range of tumors, including gastric tumors [4], neuroendocrine tumors [5], and melanomas [6]. Recent systematic reviews have revealed a positive correlation between increased Ki-67 proliferation index (PI) and higher National Institutes of Health (NIH) risk stratification, as well as poorer survival rates at various cutoff values [7, 8]. These findings underscore the potential of Ki-67 PI as a promising prognostic indicator for GIST patients.

Computed tomography (CT) has been widely used for initial diagnoses, treatment response evaluation, and recurrence detection in GISTs for its non-invasive nature [9]. Several CT imaging features have been found to be significantly associated with Ki-67 PI and patient prognosis in GISTs [10, 11]. Recent advancements in computer vision and pattern recognition have exhibited the capability of radiomics to reflect tumor heterogeneity and offer essential complementary information for diagnosis and outcome prediction, in addition to clinical and histopathologic clues [12]. Radiomics-based prediction models have also been shown to be effective in the risk stratification of GISTs. Previous research showed that a nomogram based on radiomics features extracted from portal-phase contrast-enhanced CT images outperformed the clinicopathological nomogram for predicting recurrence-free survival in GIST patients [13]. Contrast-enhanced CT was usually preferred over non-contrast-enhanced CT for radiomics feature extraction, as it was the modality of choice to characterize an abdominal mass [14]. However, a recent study found that radiomics signatures from non-contrast-enhanced CT performed similarly to those from contrast-enhanced CT in predicting NIH risk stratification, although the rationale behind this remains unexplained [15]. In this study, we evaluated and analyzed radiomics models based on non-contrast-enhanced CT and different phases of contrast-enhanced CT in predicting Ki-67 PI among patients with pathologically confirmed GISTs, using a multi-vendor approach.

## Materials and methods

### Subjects

The study was approved by the review board of Renji Hospital and the need for written informed consent was waived. This is a retrospective single-center study conducted at the Department of Radiology in Renji Hospital, using consecutive patients diagnosed with pathologically confirmed GIST from July 2012 to June 2022. The inclusion criteria were as follows: (1) CT images were available before pathological analysis, including at least one non-contrast-enhanced and three contrast-enhanced phases in a single CT examination, and (2) tumors were pathologically confirmed as GIST by fine needle aspiration or surgical specimens. The exclusion criteria consisted of (1) comorbidity of primary tumors other than GIST, (2) lacking Ki-67 PI in immunohistochemistry data, and (3) tumor that were either partially included in CT images or had suboptimal CT imaging quality. The most recent CT scan was selected if multiple contrast-enhanced CT images were available for a single subject. This study adheres to the Image Biomarker Standardisation Initiative (IBSI) guidelines for the standardization of image acquisition and analysis.

### CT image acquisition

All subjects underwent a non-contrast-enhanced abdominal CT scan and a three-phase contrast-enhanced CT scan using one of the specified CT systems, with the scanning and reconstruction parameters used in daily clinical practice. The detailed CT protocols are presented in Table S1. The iodine contrast agent was administered at a dose of 1 mL/kg of the patient’s weight, and the flow rate was 2.5–3.5 mL/s. The three phases in the contrast-enhanced CT images included arterial phase (25–30 s after injection), venous phase (70–80 s after injection), and delayed phase (180–240 s after injection).

### *CT image segmentation*

All CT images were imported to 3D Slicer (version 5.0.3, https://www.slicer.org/) for delineation. The delineated areas included the tumor lesion as much as possible without including the surrounding normal tissues or other tissues to generate a volume of interest (VOI). Interpolation (“Fill between slices”) and smoothing tool were utilized in order to make the VOI boundary smooth and accurately match the real lesion. CT image segmentation was conducted by one radiologist (Z.X.) with 3 years of radiological experience, delineated either at the delayed phase or the phase with the clearest tumor boundary in the contrast-enhanced CT. Subsequently, a simple transformation, including translation, rotation, resizing along the axial axis, or a combination of these, was manually applied to align the delineated VOI with the lesion in the non-contrast-enhanced phase and other contrast-enhanced CT phases. In rare cases, where the tumor shape was distinct in different phases, manual segmentation was performed in all four CT phases without transformation. The image segmentation process was repeated 2 months later by the same radiologist (Z.X.) on a randomly selected subset of 20 patients, and another radiologist (W.Z.) with 5 years of radiological experience performed image segmentation once on the same subset. Additional details on the conversion to mask was described in the Supplementary Materials. Two-way mixed effects, consistency, single rater/measurement intra- and inter-observer correlation coefficient were then applied. Intra- and inter-observer correlation coefficient estimates were calculated using Python software, version 3.10.8 (Python Software Foundation) based on a single-rater, absolute-agreement, two-way random-effects model [16]. The features with an intra- and inter-observer correlation coefficient greater than 0.75 were then selected for analysis.

### Training and validation dataset

All CT images were obtained using CT scanners from the following vendors: GE (*n* = 218), Philips (*n* = 19), Siemens (*n* = 96), Toshiba (*n* = 29), and UIH (*n* = 21). CT images obtained from GE scanners were used as the training dataset. Two validation datasets were created to address the imbalanced sample size of the vendors: one from Siemens (validation dataset 1), and the other from the remaining vendors (*n* = 69, validation dataset 2). These validation datasets were reserved and remained untouched until the model was fine-tuned.

### Feature selection

A total of 107 radiomics features were extracted from the VOI of each phase of CT images without applying additional filters. After filtering based on intra- and inter-observer correlation coefficient, 79 features were selected for use in model fine-tuning. These features, which included first-order statistics, shape, grey-level co-occurrence matrix (GLCM), grey-level dependence matrix (GLDM), grey-level run-length matrix (GLRLM), grey-level size-zone matrix (GLSZM), and neighboring grey tone difference matrix (NGTDM), were extracted using PyRadiomics, version 3.0.1 in Python software, version 3.10.8 (Python Software Foundation), compliant to IBSI definitions. Details about the radiomics extraction workflow are included in the Supplementary Materials. To avoid over-fitting, a limited number of radiomics features with higher predictive performance to the target variable were selected. Through exploratory data analysis, we found a high inter-feature correlation among most radiomics features. Thus, we first ranked all radiomics features based on their Spearman correlation coefficient with the value of Ki-67 PI, and included the top 4 features with inter-feature correlation coefficient maintained less than 0.9. This process was repeated for non-contrast-enhanced CT and all phases of contrast-enhanced CT.

### Model fine-tuning

The random forest models were trained for each phase of CT images to predict tumors with a high Ki-67 PI (Ki-67 > 10%). The optimization of the random forest models was performed using the Bayesian optimization algorithm in a given parameter search space. The hyperparameters with the best average area under receiver operating characteristic curve (AUC) over three-fold cross-validation were used as the optimal parameters to fit the entire training set. This process was repeated for both the non-contrast-enhanced phase and all three contrast-enhanced phases. The binary prediction was directly made by the random forest models, and the predictive performance of the models was then evaluated on both validation datasets.

### Statistical analysis

Descriptive statistics were presented as frequencies (*n*) and percentages (%) for categorical variables and mean ± standard deviation for continuous variables. Comparisons of categorical variables between different groups were performed using Fisher’s exact test or Pearson’s *χ*^2^ test with Yate’s correction for continuity. Continuous variables were compared using either Students’ *t* test or one-way analysis of variance (ANOVA), as appropriate. The DeLong test was used to calculate the variance of AUC and to compare the performance of different models. Randomized permutation test with 1000 permutations was used to determine the significance of the model predictions. A two-sided *p* value of < 0.05 was considered statistically significant. All statistical analyses were performed with Python software, version 3.10.8 (Python Software Foundation). The source code for our paper was publicly available at https://github.com/heyrict/gist-eus-open. Data in our paper was available upon reasonable request to the corresponding author.

## Results

**Fig. 1:**
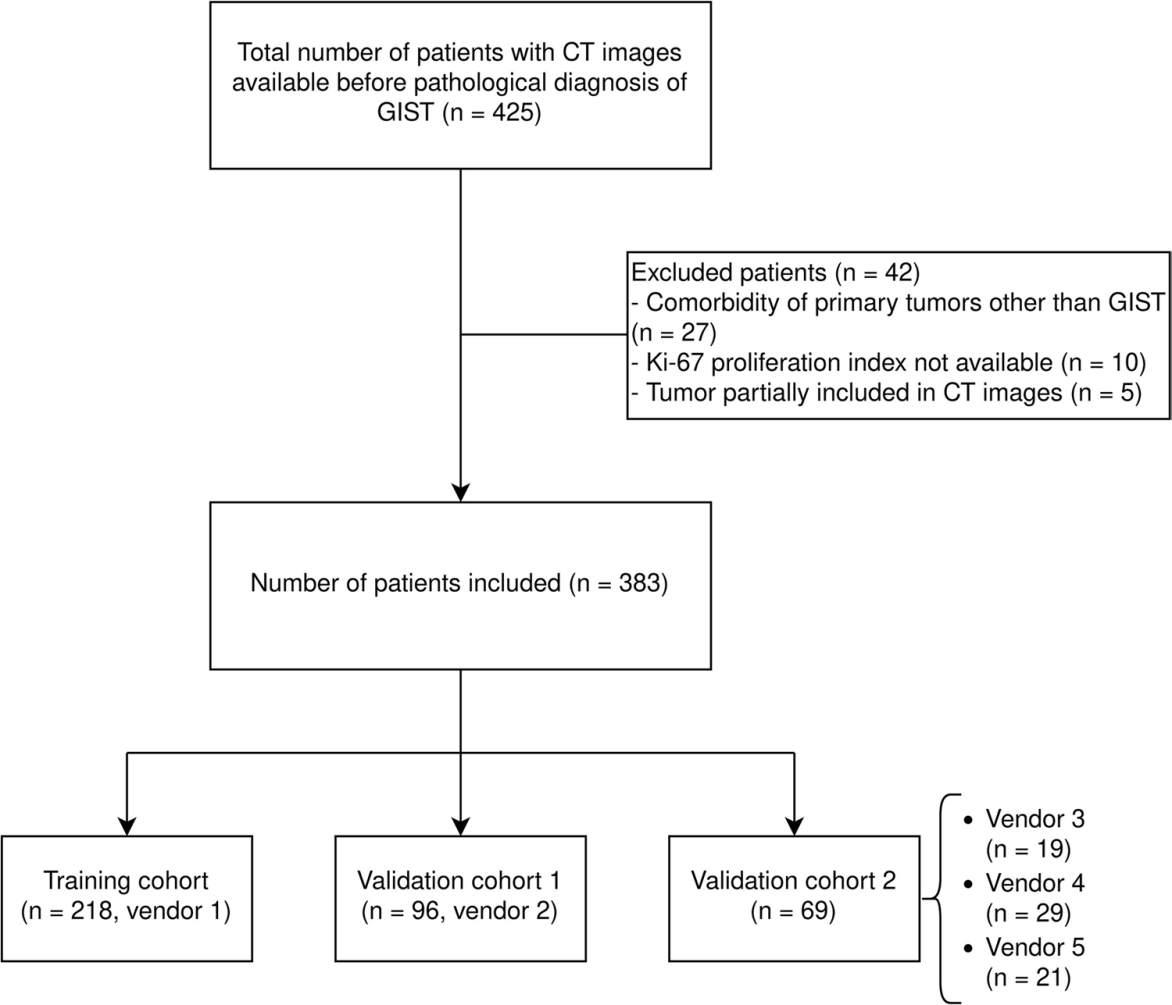
The flowchart of patient inclusion. CT, computed tomography; GIST, gastrointestinal stromal tumor

**Fig. 2:**
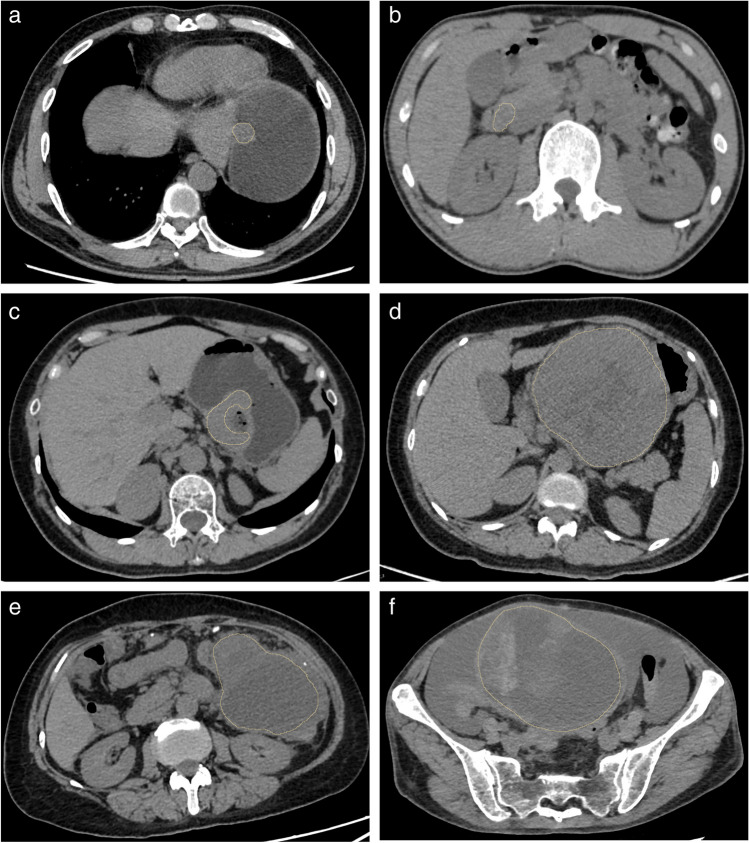
Non-contrast-enhanced CT images of patients with GISTs ordered by NGTDM Busyness. **a** Male, 51 years, Ki-67 PI 5%, NGTDM Busyness 9.67; **b** male, 27 years, Ki-67 PI 5%, NGTDM Busyness 101.66; **c** female, 59 years, Ki-67 PI 30%, NGTDM Busyness 1010.43; **d** female, 60 years, Ki-67 PI 10%, NGTDM Busyness 5823.63; **e** female, 61 years, Ki-67 PI 20%, NGTDM Busyness 13912.72; **f** female, 68 years, Ki-67 PI 30%, NGTDM Busyness 29542.20. NGTDM, neighboring grey tone difference matrix; PI, proliferation index

From July 2012 to June 2022, a total of 383 patients were retrospectively included in the study (Fig. 1). These patients were then separated into training set (*n* = 218), validation set 1 (*n* = 96), and validation set 2 (*n* = 69). The baseline characteristics of the patients are shown in Tables 1 and 2. There was no statistical significance in age (*p* = 0.627), gender (*p* = 0.570), tumor diameter (*p* = 0.461), Ki-67 PI level (*p* = 0.307), and contrast enhancement ratio (*p* = 0.971) among different datasets. The proportion of the high Ki-67 PI group was 12.39% (27 of 218), 9.38% (9 of 96), and 17.39 (12 of 69) for the training set, validation set 1, and validation set 2, respectively. Tumor size was significantly larger in the high Ki-67 PI group than the low Ki-67 PI one in all datasets (*p* < 0.01). The contrast enhancement ratio, calculated by dividing the CT attenuation value obtained at the maximum-enhanced phase by that obtained during the non-contrast-enhanced phase, was lower in both the high Ki-67 PI group in training set (*p* = 0.042) and validation set 1 (*p* = 0.030). No statistical significances were found in age (*p* = 0.096, 0.615, 0.183 for training set, validation set 1, and validation set 2, respectively) and gender (*p* = 0.310, 0.181, 1.000 for training set, validation set 1, and validation set 2, respectively) between low and high Ki-67 PI groups. Representative non-contrast-enhanced CT images and their segmentation borders are shown in Fig. 2.

**Table 1:** Baseline characteristics of patients in training and validation datasets

	Training (*n* = 218)	Validation 1 (*n* = 96)	Validation 2 (*n* = 69)	*p*
Age (years)	60.91 ± 11.94	62.38 ± 12.71	61.33 ± 13.07	0.627
Gender				0.570
Female	110 (50.46%)	43 (44.79%)	36 (52.17%)	
Male	108 (49.54%)	53 (55.21%)	33 (47.83%)	
Diameter (mm)	55.01 ± 40.64	54.96 ± 41.08	61.80 ± 42.43	0.461
Ki-67 PI				0.307
Ki-67 ≤ 10%	191 (87.61%)	87 (90.62%)	57 (82.61%)	
Ki-67 > 10%	27 (12.39%)	9 (9.38%)	12 (17.39%)	
CER (%)	2.26 ± 0.81	2.27 ± 0.77	2.24 ± 0.76	0.971

*CER*, contrast enhancement ratio; *PI*, proliferation index

**Table 2:** Baseline characteristics of GIST patients stratified by Ki-67 proliferation index in different datasets

	Training			Validation 1			Validation 2		
	Low Ki-67 PI (*n* = 191)	High Ki-67 PI (*n* = 27)	*p*	Low Ki-67 PI (*n* = 87)	High Ki-67 PI (*n* = 9)	*p*	Low Ki-67 PI (*n* = 57)	High Ki-67 PI (*n* = 12)	*p*
Age (years)	61.42 ± 11.70	57.33 ± 13.25	0.096	62.59 ± 13.24	60.33 ± 5.24	0.615	62.30 ± 13.14	56.75 ± 12.17	0.183
Gender			0.310			0.181			1.000
Female	99 (51.83%)	11 (40.74%)		41 (47.13%)	2 (22.22%)		30 (52.63%)	6 (50.00%)	
Male	92 (48.17%)	16 (59.26%)		46 (52.87%)	7 (77.78%)		27 (47.37%)	6 (50.00%)	
Diameter (mm)	51.68 ± 39.77	78.55 ± 39.60	0.001	51.28 ± 39.51	90.52 ± 41.03	0.006	55.79 ± 38.18	90.36 ± 51.32	0.009
CER (%)	2.30 ± 0.82	1.96 ± 0.70	0.042	2.33 ± 0.78	1.75 ± 0.36	0.030	2.32 ± 0.79	1.88 ± 0.50	0.070

*CER*, contrast enhancement ratio; *PI*, proliferation index

**Fig. 3:**
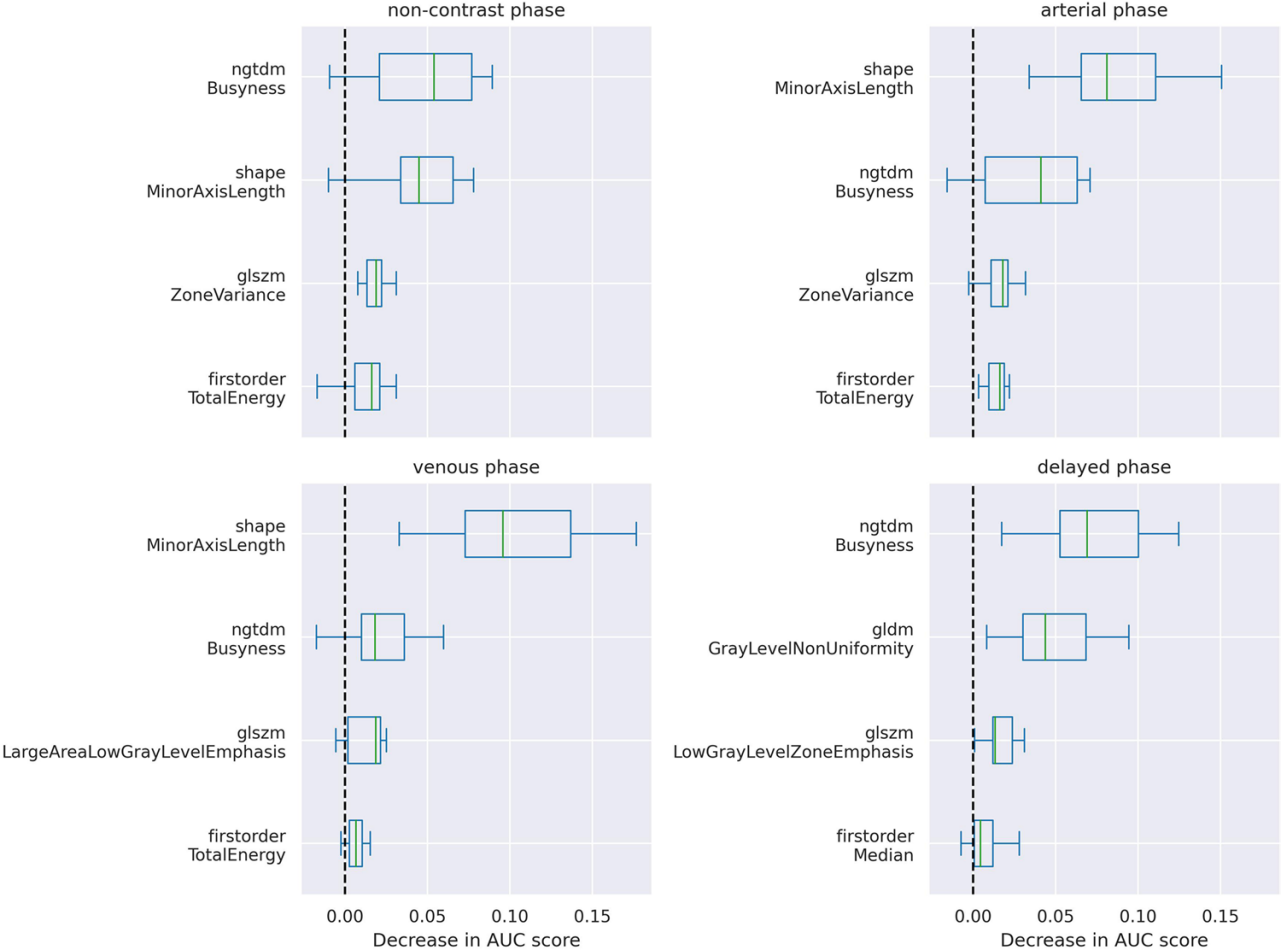
Permutation importance of radiomics features in models trained with non-contrast-enhanced CT images or different phases of contrast-enhanced CT images. The left vertical coordinates indicate the radiomics features; the horizontal coordinates indicate the decrease of AUC value after random permutation of the exact feature. AUC, area under the receiver operating characteristic curve

After feature selection, four radiomics features were selected for each phase and were used in the model optimization pipeline. As is shown in Fig. 3, similar features were selected from different phases of CT images. NGTDM Busyness was selected and made a significant contribution in all four phases, while the minor axis length of tumor was selected in most phases except the delayed phase. Both GLSZM Zone variance and total energy in the first-order statistics were selected twice in total.

**Fig. 4:**
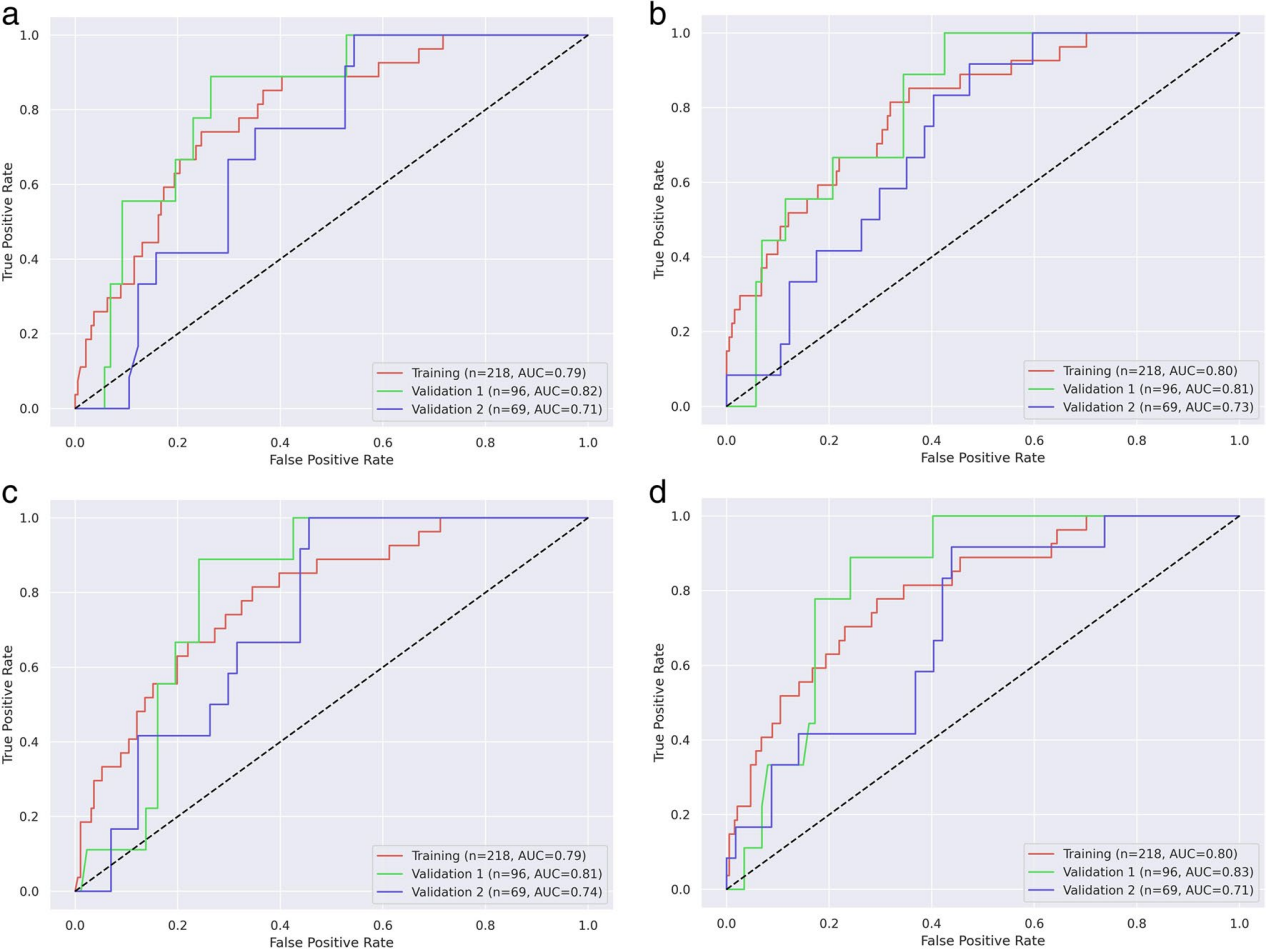
Comparison of receiver operating characteristic curve in a model trained with non-contrast-enhanced CT images alone and different phases of contrast-enhanced CT images. **a** Model trained with non-contrast-enhanced CT images; **b** model trained with arterial phase on contrast-enhanced CT images; **c** model trained with venous phase on contrast-enhanced CT images; **d** model trained with delayed phase on contrast-enhanced CT images. AUC, area under the receiver operating characteristic curve

The performances of each model were evaluated using AUC, accuracy, sensitivity, and specificity, as shown in Table 3 and Fig. 4. The AUC values for non-contrast-enhanced phase and three contrast-enhanced phases (arterial, venous, delayed phase) were 0.822 (*p* = 0.001, 95%CI 0.707–0.938), 0.814 (*p* = 0.001, 95%CI 0.700–0.927), 0.807 (*p* = 0.001, 95%CI 0.708–0.905), and 0.834 (*p* = 0.001, 95%CI 0.740–0.928) respectively in validation set 1. The AUC values for non-contrast-enhanced phase and three contrast-enhanced phases were 0.711 (*p* = 0.009, 95%CI 0.580–0.842), 0.725 (*p* = 0.005, 95%CI 0.594–0.856), 0.737 (*p* = 0.004, 95%CI 0.613–0.860), and 0.709 (*p* = 0.012, 95%CI 0.560–0.857) respectively in validation set 2. No significant differences were found with the DeLong test in receiver operating characteristic curves between either two of these models, in both validation sets 1 (*p* = 0.616 to 0.918) and 2 (*p* = 0.624 to 0.981). The Radiomics Quality Score (RQS 1.0) [17] of our study is 10 (details in Supplementary Material).

**Table 3:** Predictive performance of radiomics models in non-contrast-enhanced and different contrast-enhanced phases

Dataset	Metric	Non-contrast	Arterial	Venous	Delayed
Training	AUC	0.792	0.803	0.791	0.801
	Accuracy	0.739	0.757	0.752	0.743
	Sensitivity	0.741	0.667	0.667	0.704
	Specificity	0.738	0.770	0.764	0.749
Validation 1	AUC	0.822	0.814	0.807	**0.834**
	Accuracy	0.708	0.760	0.771	0.688
	Sensitivity	0.889	0.667	0.667	0.889
	Specificity	0.690	0.770	0.782	0.667
Validation 2	AUC	0.711	0.725	**0.737**	0.709
	Accuracy	0.667	0.696	0.696	0.609
	Sensitivity	0.667	0.500	0.500	0.500
	Specificity	0.667	0.737	0.737	0.632

Best AUC values in each validation dataset were marked with bold text. *AUC*, area under the receiver operating characteristic curve

## Discussion

In this study, we evaluated and analyzed the radiomics models based on non-contrast-enhanced CT and different phases of contrast-enhanced CT in predicting Ki-67 PI. The results showed that the radiomics models trained on different CT images had good capabilities of predicting Ki-67 PI of GISTs in both validation datasets, regardless of CT scanners’ vendor and protocol. The predictive performance of the radiomics model based on non-contrast-enhanced CT images was similar to those based on contrast-enhanced CT images, indicating that the radiomics models have great potential for identifying the Ki-67 proliferation status in GISTs without contrast material and have a good generalizability across vendors. Interestingly, our study found several relevant radiomics features that were repeatedly selected and had good predictive value across non-contrast-enhanced CT and different phases of contrast-enhanced CT images, which could possibly explain for the non-inferiority of the radiomics model based on non-contrast-enhanced CT.

Recently, the radiomics features have been utilized to demonstrate significant associations with risk grading [13, 18, 19] and Ki-67 PI classification [20, 21] in GISTs. In comparison to previous studies targeting Ki-67 PI, our radiomics model achieved comparable AUC values in the validation datasets (0.735 and 0.820 vs. 0.784 to 0.828). Arterial phase [22], venous phase [13, 18], or a combination of different phases [19, 23] was commonly used in extracting the radiomics features in GISTs. Recently, one study has found similar AUC values (0.941 vs. 0.899) when comparing the radiomics models trained on non-contrast-enhanced CT and contrast-enhanced CT images in the task of risk stratification with NIH criteria [15]. Similarly, our study did not find significant differences among radiomics models trained on non-contrast-enhanced phase and different phases of contrast-enhanced CT images in predicting Ki-67 PI.

The phenomenon that the radiomics model based on non-contrast-enhanced CT images performed similarly to their contrast-enhanced CT counterparts have been reported in several studies previously in pulmonary neoplasms. One study comparing the radiomics models in non-small cell lung cancer diagnosis of pulmonary nodules showed that quantitative texture features performed similarly with or without contrast-enhanced images [24]. Another recent study has demonstrated comparable results in the radiomics models for predicting the epidermal growth factor receptor (EGFR) mutation status in non-small cell lung cancers using general radiomics features, defined as features without significant differences between CT images with or without contrast enhancement [25]. The results also indicated no statistically significant differences between the performance of the radiomics models trained on non-contrast-enhanced and contrast-enhanced CT images. Although this is still unproven, it was hypothesized that the radiomics signature in pulmonary nodules may be independent of the injection of a contrast agent as the selected radiomics features measure the correlation, uniformity, or deviation of the pixels, which reduces the effect of contrast increase of the overall image intensity [24]. This hypothesis may also extend to GIST lesions. Moreover, the results of our study solidified the hypothesis as we observed comparable performances in the radiomics models trained on various phases of contrast-enhanced CT images in addition to non-contrast CT images.

In our exploratory analyses, we found a high inter-feature correlation among most radiomics features during the feature extraction procedure and thus introduced a simple method to prune over-correlated features. This procedure allowed us to obtain a more compact size of features compared with most radiomics studies, without a significant impact on the performance. One important finding of our study was that the most relevant radiomics features maintain their predictive value regardless of contrast agent and CT imaging phases. The feature NGTDM Busyness was selected and showed great feature importance in both non-contrast-enhanced and all three phases of contrast-enhanced CT images. This feature has also been noted in previous studies as an imaging marker representative of Ki-67 PI in GISTs [20, 21]. Minor axis length, a representative feature of the tumor size, was selected in all CT images except those in the delayed phase of contrast-enhanced CT. This is not surprising as tumor size has been extensively used to predict the malignancy of GISTs, and is included in multiple risk stratification systems, including the most famous NIH criteria [1, 26]. The reason that these features have not always achieved high ranks in other studies could potentially be attributed to the high inter-feature correlation among different radiomics features. In conclusion, our findings suggest that NGTDM Busyness and tumor size are crucial in predicting Ki-67 PI, regardless of contrast agent or CT imaging phases. NGTDM Busyness may serve as a phase-agnostic texture feature stable to contrast agents in GISTs. This could probably explain the non-inferiority of the radiomics model trained solely on non-contrast-enhanced CT images in comparison to those with contrast enhanced CT images.

### Study limitations

Our study has several limitations. Firstly, our study was a single-center retrospective study with a limited sample size. To minimize selection bias, we utilized a multi-vendor approach in validating our models. In addition, there was a gap between AUC values in the two validation datasets, which may result from the limited sample size, or possibly a scanner dependency problem. Although we are confident in the generalizability of our models as the DeLong test is not significant and all of them achieved an AUC > 0.7, a larger, prospective, multi-center trial is still needed to confirm these results. Secondly, the sample size of CT images obtained from different vendors was unevenly distributed; thus, we combined images from several minor vendors into one validation dataset to mitigate potential biases. In addition, the Ki-67 PI of several samples were acquired from fine needle aspiration specimens, which could lead to potential bias considering the heterogeneity in Ki-67 PI values. Nevertheless, a previous study has demonstrated good correlation between FNA-based Ki-67 PI and mitotic count in surgical specimens, and we have determined that fine needle aspiration can be used for Ki-67 PI assessment in GISTs [27]. Lastly, our use of three-dimensional VOI for tumor segmentation is time-consuming and subject to debate regarding inter-reader variability. However, previous research has shown that three-dimensional texture analysis offers a more comprehensive representation of tumor heterogeneity in abdominal tumors [28], and we achieved an acceptable intra- and inter-observer correlation in our radiomics features (79 over 107 features with both intraclass correlation coefficient > 0.75).

## Conclusions

Our study showed that a radiomics model based on non-contrast-enhanced CT images is not inferior to those trained on different phases of contrast-enhanced CT images in predicting the Ki-67 PI in GISTs. Several radiomics features, including NGTDM Busyness as well as minor axis length of GIST, have crucial predictive value in predicting Ki-67 PI regardless of contrast agent administration or CT imaging phases. Our results suggest that GIST may exhibit similar radiological patterns across different phases of contrast-enhanced CT images irrespective of the timing after bolus injection, and such radiomics features may help quantify these patterns to predict risk stratification or Ki-67 PI when used for modeling purposes.

### Supplementary Information

ESM 1 (PDF 185 kb)

ESM 2 (PDF 18 kb)

## Abbreviations

ANOVA: Analysis of variance
AUC: Area under the receiver operating characteristic curve
CT: Computed tomography
EGFR: Epidermal growth factor receptor
GISTs: Gastrointestinal stromal tumors
GLCM: Grey-level co-occurrence matrix
GLDM: Grey-level dependence matrix
GLRLM: Grey-level run-length matrix
GLSZM: Grey-level size-zone matrix
IBSI: Image Biomarker Standardisation Initiative
NGTDM: Neighboring grey tone difference matrix
NIH: National Institutes of Health
PI: Proliferation index
RQS: Radiomics Quality Score
VOI: Volume of interest

## Declarations

### Guarantor

The scientific guarantor of this publication is Yan Zhou.

### Statistics and biometry

One of the authors, Yang Song, has significant statistical expertise.

### Study subjects or cohorts overlap

Some study subjects or cohorts have been previously reported in Zhang Q W, Gao YJ, Zhang R.Y, Zhou XX, Chen SL, Zhang Y, Liu Q, Xu JR, Ge ZZ (2020) Personalized CT-based radiomics nomogram preoperative predicting Ki-67 expression in gastrointestinal stromal tumors: a multicenter development and validation cohort. *Clin Trans Med* 9, 12. 10.1186/s40169-020-0263-4.

### Methodology

• retrospective

• observational

• performed at one institution
