# Supplementary material for: Prediction of high Ki-67 proliferation index of gastrointestinal stromal tumors based on CT at non-contrast-enhanced and different contrast-enhanced phases
Source: Eur Radiol. 2023 Sep 29;34(4):2223–32. doi: 10.1007/s00330-023-10249-3 (PMC10957607; doi:10.1007/s00330-023-10249-3)
Supplement: Supplementary file 1 — (PDF 185 kb) [file 330_2023_10249_MOESM1_ESM.pdf]

Prediction of high Ki-67 proliferation index of gastrointestinal stromal tumors based on CT at non-contrast-enhanced and different contrast-enhanced phases

Electronic Supplementary Material

Detailed radiomics extraction workflow on feature selection

GIST lesions were segmented as planar contour with 3d Slicer (version 5.0.3, https://www.slicer.org/). The delineated lesions by one radiologist (ZX) were converted to binary labelmap in 3d Slicer and exported to standard NRRD format. The delineated lesions by another radiologist (WZ) used for inter-observer correlation were exported as 3d volume node stored in NRRD format. Original DICOM files were first converted to NRRD formats with 3d Slicer as some vendors use Multi-volume data which was difficult to process, and then read as SimpleITK (version 2.2.1) images. The segmentation geometry was corrected using slicerio (version 0.1.8) if exported as 3d volume node, and the segmentations were then converted SimpleITK images. Before extraction of radiomic features, the segmentations were first resampled to the image geometry using nearest neibour interpolation. No image normalization, resampling and discretization were done as spatial resolution acquired were similar among different vendors (approximately 0.8mm x 0.8mm in-plane resolution; 1.0cm or 1.25cm slice thickness) and we used a multi-vendor approach to validate our models. Texture features were extracted by slice along axial plane which has the highest in-plane resolution. No additional filters were applied to avoid over-fitting, as we found a high inter-feature correlation among most radiomics features during our exploratory data analysis. A total of 107 features, including 18 features of first order statistics, 14 features of shape, 24 features of grey-level co-occurrence matrix (GLCM), 14 features of gray-level dependence matrix (GLDM), 16 features of grey-level run-length matrix (GLRLM), 16 features of grey-level size-zone matrix (GLSZM) and 5 features of neighbouring gray tone difference matrix (NGTDM), were extracted using PyRadiomics on Python software, version 3.10.8 (Python Software Foundation). The default texture paramters from PyRadiomics were used for feature computation. Explicitly, GLCM was calculated by symmetric co-occurrence matrices with a distance of 1. GLSZM was calculated with a linkage distance of 1. NGTDM was calculated with a neighbourhood distance and distance norm of 1.

Supplementary Table S1: Vendors and protocols of CT images for patients with GISTs.

Vendor	GE	Siemens	Philips	UIH	Toshiba
Scanner	LightSpeed VCT, Revolution CT, Revolution GSI, Optima CT680 Series	SOMATOM Force, SOMATOM Definition AS+	IQon - Spectral CT	uCT 760, uCT 780, uCT 960+	Aquilion, Aquilion ONE
Tube voltage (kV)	120	120	120	120	120
Tube current (mA)	160 - 455	500 or 600	265 - 270	270 - 600	300 or 380
Rotation time (s)	0.8	0.5	0.5	0.5	0.5
Detector collimation (mm)	64 × 0.625	64 × 0.6 or 96 × 0.6	64 × 0.625	80 × 0.5 or 120 × 0.5	80 × 0.5
Pitch	1.0 or 1.375	0.6 or 1.0	1	1.0 or 1.2	0.813 or 0.844
Slice thickness (mm)	1.25	1	1	1.0 or 1.25	1
Matrix size	512 × 512	512 × 512	512 × 512	512 × 512	512 × 512
FOV (mm)	360, 412, 429 or 500	372 or 378	417	345, 384 or 410	347 or 402
Algorithm (B)	standard	standard	standard	standard	standard
